# Genome sequences of dengue virus serotypes 2, 3, and 4 isolated from adult patients in Thailand

**DOI:** 10.1128/mra.00522-24

**Published:** 2024-10-29

**Authors:** Adisak Songjaeng, Kwanrutai Chin-Inmanu, Phitchapha Proykhunthod, Ranyikar Poraha, Preeyanuch Sayboonruan, Dararat Prayongkul, Dumrong Mairiang, Yupin Suputtamongkol, Nasikarn Angkasekwinai, Nuntaya Punyadee, Panisadee Avirutnan

**Affiliations:** 1 Division of Dengue Hemorrhagic Fever Research, Faculty of Medicine Siriraj Hospital, Mahidol University, Bangkok, Thailand; 2 Siriraj Center of Research Excellence in Dengue and Emerging Pathogens, Faculty of Medicine Siriraj Hospital, Mahidol University, Bangkok, Thailand; 3 Division of Medical Bioinformatics, Research Group and Research Network Division, Research Department, Faculty of Medicine Siriraj Hospital, Mahidol University, Bangkok, Thailand; 4 Molecular Biology of Dengue and Flaviviruses Research Team, Medical Molecular Biotechnology Research Group, National Center for Genetic Engineering and Biotechnology, National Science and Technology Development Agency, Bangkok, Thailand; 5 Department of Medicine, Faculty of Medicine Siriraj Hospital, Mahidol University, Bangkok, Thailand; Katholieke Universiteit Leuven, Leuven, Belgium

**Keywords:** dengue genome, adult patient, ivermectin, DENV evolution

## Abstract

Here, we present the genome sequences of dengue viruses (DENV) isolated from adult patients in Thailand during 2016–2017: DENV2 (412749), DENV3 (416384), and DENV4 (416709). These sequences provide valuable genetic and evolutionary information for dengue research and antiviral development.

## ANNOUNCEMENT

Dengue virus (DENV) infection remains a significant global health concern, causing a spectrum of illness from mild dengue fever (DF) to life-threatening dengue hemorrhagic fever (DHF) and dengue shock syndrome (DSS) (1). DENV, a member of the family *Flaviviridae*, possesses a positive single-stranded RNA genome (approximately 10.7 kb), with four distinct serotypes. Understanding the genetic background and pathogenesis of DENV is crucial for research, especially for clinical trials and vaccine development. Here, we present representative sequences of DENV from patients enrolled in a clinical trial in Thailand (Clinicaltrials.gov ID: NCT02045069) (2).

The dengue viruses [DENV2 (412749); DENV3 (416384); and DENV4 (416709)] were isolated using C6/36 mosquito cell cultures (3), from baseline (pre-treatment) plasma samples of adult patients in the trial with ivermectin. The metadata of each sample is shown in Table 1. The isolated viruses were further propagated in C6/36 cells for three passages. Six days post-infection, the culture supernatant was harvested for RNA extraction using the MagLead 12gC system and MagDEA Dx SV kit (Precision System Sciences Co., Ltd, Japan). cDNA was constructed using Random Hexamer primers and ProtoScript II First Strand cDNA Synthesis Kit (New England Biolabs, USA). Eleven overlapping amplicons (approximately 1.2 kb in size), were amplified using a PCR tiling method with serotype-specific primers and Q5 High-Fidelity DNA polymerase (New England Biolabs, USA). The specific primers were designed using PrimalScheme software (4) with reference sequences obtained from the GenBank database. The sequences of primers are provided in GitHub (https://github.com/si-medbif/virus_seq/tree/main/Dengue_Virus/DENV_multiplex_tiling_PCR/). All PCR fragments were pooled into a single tube per sample before being ligated with the same barcode. Samples given unique barcodes were combined before sequencing. Viral sequences were determined using the Oxford Nanopore Technology platform [Siriraj Long read Lab (Si-LoL), Faculty of Medicine Siriraj Hospital, Mahidol University]. Sequencing libraries were prepared using the Rapid Barcoding sequencing kit (SQK-RBK114-24). Briefly, rapid barcodes were added to samples, pooled, and loaded onto an R10.4.1 flow cell. The sequencing was performed on the GridION Mk1 for 72 h. The basecalling process was carried out using MinKNOW v5.5.5 with the super-accurate base calling model. Sequencing data were analyzed using the ARTIC bioinformatics pipeline v1.2.3 (5). In brief, each read was demultiplexed to its respective sample, and primers/adapters region removed using Guppy v6.5.7. Reads were filtered with ARTIC guppyplex tools to retain those approximately 400–1,700 bp. Consensus sequences were then generated using Medaka v1.11.3 (6), with primer excluded prior to assembly.

**TABLE 1:** Patient information, sample collection details, and sequencing data for DENV isolates with comparison to closest GenBank sequences

Description	Sample ID		
	412749	416384	416709
Sample information			
Collection date	6/1/2016	28/5/2017	12/6/2017
Sex	Male	Male	Male
Age (years)	22	24	20
Dengue serotype (qRT-PCR)	DENV2	DENV3	DENV4
Day of illness	3	2	5
Type of infection	Secondary	Secondary	Secondary
Disease severity (WHO 1997)	DHF	DF	DF
Disease severity (WHO 2009)	Dengue with warning signs	Dengue without warning signs	Dengue without warning signs
Viral genome (Log10 copies/mL)	4.94	6.21	5.66
NS1 level (ng/mL)	1,868.12	43.19	128.77
Sequencing data			
GenBank accession no.	PP774214	PP774215	PP774216
SRA accession no.	SRX24762395	SRX24762396	SRX24762397
Total number of reads	1,994	1,947	1,617
Total number of bases	1,692,298	1,855,746	1,421,638
*N*_50_ value	951	1,107	1,027
Lengths of obtained genome (bp)	10,703	10,692	10,626
Accession no. for closely related sequences (% identity)	KY672945 (99%)	LC410193 (99%)	MG601754 (99%)

**Fig 1:**
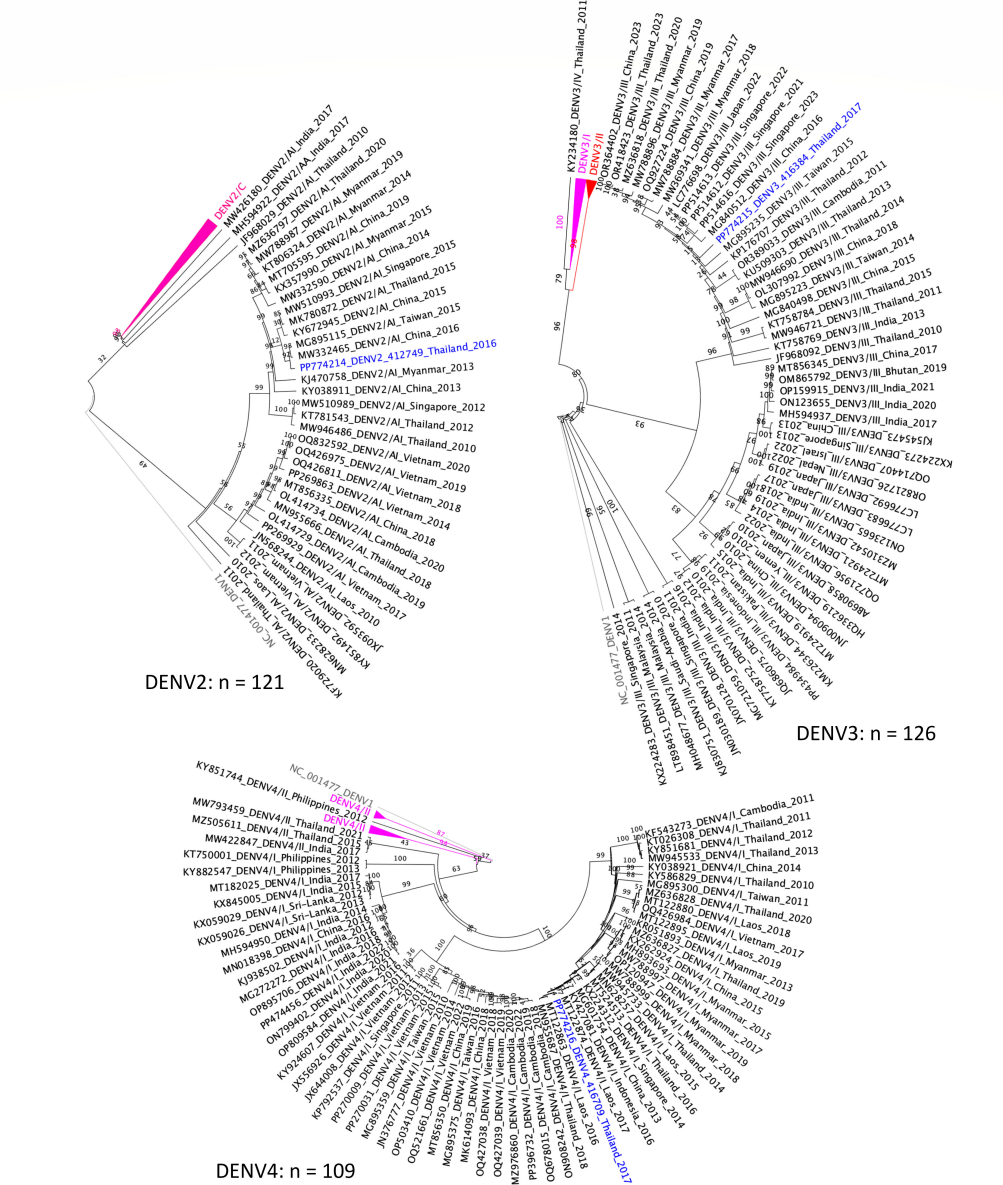
Maximum likelihood phylogenetic trees of dengue virus (DENV) serotypes 2, 3, and 4. Trees were constructed using envelope (E) gene sequences from 121 DENV2, 126 DENV3, and 109 DENV4 isolates (2010–2023) from the NextStrain database, with 1,000 bootstrap replicates. DENV isolates from the current study are indicated by blue text: DENV2 (412749), DENV3 (416384), and DENV4 (416709). Each isolate clusters within its respective genotype.

A maximum likelihood phylogenetic tree was constructed with IQ-TREE (7) using envelope (E) gene sequences from Asian DENV samples collected between 2010 and 2023, sourced available from the NextStrain database. The E gene sequences were aligned using MUSCLE v5.1 (8) with default settings before phylogenetic analysis. Results revealed that 412749, 416384, and 416709 were classified into the DENV2/Asian I, DENV3/III, and DENV4/I genotypes, respectively (Fig. 1). These assignments were verified by the Dengue Virus Typing tool (9, 10), which confirmed the phylogenetic analysis.

## Data Availability

The complete genome sequences of the three DENV clinical strains, DENV2 (412749), DENV3 (416384), and DENV4 (416709), are available in the NCBI GenBank database under accession numbers PP774214, PP774215, and PP774216, respectively. The raw sequencing data of three samples were deposited in the Sequence Read Archive (SRA) database (SRA accession numbers SRX24762395, SRX24762396, and SRX24762397) under the BioProject accession number PRJNA1118493.
